# Longitudinal Associations Between Adolescent Dating Violence Victimization and Adverse Outcomes: A Systematic Review

**DOI:** 10.1177/15248380231174504

**Published:** 2023-05-25

**Authors:** Laura Campo-Tena, Simon R. Larmour, Noemí Pereda, Manuel P. Eisner

**Affiliations:** 1 University of Cambridge, UK; 2 University of Barcelona, Spain

**Keywords:** dating violence, domestic violence, adolescent victims, sexual assault, mental health and violence

## Abstract

Evidence on the outcomes of adolescent dating violence (ADV) victimization mainly derives from cross-sectional studies, which have limitations in suggesting causal relationships. Furthermore, the complexity of factors and overlapping dimensions in dating violence research, such as the forms of violence experienced, may have contributed to the variability of findings across the literature. To address these gaps and provide a more comprehensive understanding of the impact of ADV, this study reviews findings from prospective cohort studies, with a focus on the type of violence experienced and the gender of the victim. A systematic search was conducted in nine electronic databases and additional relevant journals. Prospective longitudinal studies were included if dating violence victimization occurred during adolescence and chronologically preceded the outcomes. A quality assessment was conducted using the Mixed Methods Appraisal Tool. A narrative approach was used to synthesize findings. After screening 1,838 records, 14 publications met the selection criteria and were included in this review. Our findings suggest that experiencing ADV is longitudinally associated with many adverse outcomes, including higher internalizing symptoms and externalizing behaviors, poorer well-being, increased substance use, and increased revictimization. However, the associations are not consistently reported across studies when considering the type of ADV experienced and the gender of the victim. This review highlights the limited number of longitudinal studies examining the outcomes of ADV victimization, the unbalanced approach in investigating different forms of violence, and the lack of diverse samples examining this subject. Implications for research, policy, and practice are outlined.

## Background

Adolescent dating violence (ADV), defined as any intentional physical, sexual, or psychological violence or stalking by a current or former partner during adolescence (Centers for Disease Control & Prevention, 2017), is characterized by significantly high prevalence rates. A meta-analysis of 101 studies revealed that around one in four adolescents experience physical ADV victimization and that 14% of girls and 8% of boys are exposed to sexual ADV (Wincentak et al., 2017). However, victimization estimates vary widely across the literature. For instance, a review by Leen et al. (2013) found that prevalence rates for psychological/emotional violence range broadly—from 17% to 88% for girls and from 24.4% to 85% for boys. A more recent review on the prevalence of ADV in Europe reiterated the heterogeneity in the prevalence rates of different forms of victimization (Tomaszewska & Schuster, 2021). This variability has been attributed to the diverse interconnecting variables, such as the various age ranges of participants included and the multiple sampling and measurement methods employed in these studies, but also to the different subtypes of violence measured (Ocampo et al., 2007; Tomaszewska & Schuster, 2021). Furthermore, the different forms of violence (e.g., physical, psychological, sexual) have been found to differentially impact the victims of ADV (Eshelman & Levendosky, 2012). Therefore, to better capture the inherent complexity of ADV and its outcomes, it is relevant to consider the full array of overlapping dimensions that the phenomenon involves, including different forms of ADV (Leadbeater et al., 2018).

The gender of the victim is another relevant aspect to consider in this context. Research points to gender differences in how individuals develop emotionally and psychologically, in the way they cope with stress, and in how they manifest the consequences of victimization (Dulmus & Hilarski, 2006; Maschi et al., 2008; Werner & Smith, 1982, 1992). This has been linked to the fact that generally boys and girls go through developmental stages at a different pace, which results in distinct gender-specific social, cultural, and psychological demands (Turner et al., 1995). For instance, a review of population-based longitudinal studies of predictors for suicidal behavior among adolescents and young adults found that dating violence was a female-specific risk factor (Miranda-Mendizabal et al., 2019). Also, research suggests that, following victimization, boys tend to exhibit more externalizing behaviors compared to girls, while girls are more prone to internalizing symptoms (Maschi et al., 2008; Dulmus & Hilarski, 2006). However, there are some mixed findings in regards to this question as other studies have suggested the contrary (e.g., Shorey et al., 2011).

### Outcomes Associated With Dating Victimization and Methodological Issues

Experiencing dating violence during adolescence has been associated with serious mental health outcomes, including internalizing and externalizing symptoms or behaviors, aside from any physical consequences. Internalizing symptoms have been frequently explored in this context. For instance, it has been documented that victims of dating violence report increased depression and anxiety (Haynie et al., 2013; Fix et al., 2021; Martz et al., 2016), suicidal behaviors (Ackard et al., 2007; Martz et al., 2016), and eating disorder behaviors (Ackard et al., 2007). Some externalizing behaviors, such as sexual risk behaviors (Alleyne-Green et al., 2016; Barros et al., 2011; Martz et al., 2016) and violent behavior (Fix et al., 2021; Holmes & Sher, 2013), have also been linked with ADV victimization. In addition, exposure to dating violence has been associated with declines in poorer self-rated health, a dimension of subjective well-being (Burton et al., 2016; Copp et al., 2016; Coker et al., 2000). Furthermore, there are lifestyle factors that may compromise the health of survivors. For instance, substance use has been consistently connected with ADV in the literature (Baker, 2016; Epstein-Ngo et al., 2013; Lormand et al., 2013; Temple & Freeman, 2011) and its use following victimization has been described as a coping mechanism by teenagers in abusive relationships (Baker, 2016). Lastly, ADV victimization has also been associated with an increased risk of revictimization, which refers to victimization occurring after the initial abuse has been reported (Barnes et al., 2009; Exner-Cortens et al., 2017; Smith et al., 2003). This highlights the importance to timely address and prevent the perpetuation of victimization among adolescents.

It is important to acknowledge that most evidence on the potential effects of dating violence derives from cross-sectional studies, where correlated variables could be both predictors and consequences. To establish stronger conclusions on associations that suggest causality, it is essential that the predicting variable precedes the outcome variable, a requirement that is met in prospective longitudinal designs (Exner-Cortens et al., 2013; Murray et al., 2009). Therefore, a comprehensive review of longitudinal studies that takes into account temporal sequencing is crucial to gain a better understanding of the impact of dating violence.

### The Current Study

This systematic review has three main objectives. First, to synthesize some methodological characteristics of the included publications and assess their quality, which will provide insights into the strength and context of the evidence supporting longitudinal associations between ADV victimization and its outcomes. Outcomes of interest are any variables related to internalizing symptoms, externalizing behaviors, substance use, well-being, and revictimization. Second, to examine whether particular forms of violence (i.e., physical, psychological, sexual) are associated differently with outcomes. And third, to explore whether the significance of these associations varies by the gender of the victim. To the best of our knowledge, this is the first comprehensive systematic review of longitudinal studies that addresses these specific research questions.

## Methods

### Study Protocol

The current study is a systematic review of prospective cohort studies. The methodology was previously documented in a protocol to minimize bias, as suggested by Cochrane (Higgins et al., 2020). The protocol was pre-registered with PROSPERO in November 2020, Registration Number: CRD42020219961. Protocol amendments were non-substantial (e.g., expanding the list of searched databases and better specifying the outcomes of interest) and documented in PROSPERO. The review process adhered to The Preferred Reporting Items for Systematic Review and Meta-Analysis (PRISMA) guidelines (Page et al., 2021; see Supplemental File).

### Eligibility Criteria

Publications were included in this review if there was explicit information confirming that the predictor (i.e., ADV victimization) occurred during adolescence (i.e., 10–19 years old, as defined by the World Health Organization) and chronologically preceded the outcome(s). Studies including samples from any country and with any cultural background, sexual orientation, and gender identity were eligible. Publications based on non-prospective longitudinal data, or that did not provide evidence on the desired chronological order of independent and dependent variables, were excluded. However, if studies also measured ADV victimization concurrently with outcomes, they were included in the review, although only findings derived from longitudinal analyses, and not cross-sectional, were considered in our analysis. Samples that did not fall within the age range of 10 to 19 years old, either partially or fully, or did not explicitly provide information on the age of the victims at the time of victimization, were excluded from this review.

### Search Strategy and Study Selection

Electronic searches for peer-reviewed and gray literature, including research reports and master’s and doctoral theses, were conducted in November and December 2020, covering the period between January 2000 and December 2020, in the following databases: Pubmed/Medline, IBSS, PsycINFO, Global Index Medicus WHO, Proquest One Literature, JSTOR, LILACS, SciELO, and Google Scholar. Several relevant Spanish journals were listed following discussion with the team members and searched for additional studies. Although no language restrictions were considered for the screening criteria, searches were mostly conducted in English, and also in Spanish in relevant databases and journals. The full search strategy is available in the Supplemental Material.

**Figure 1.**
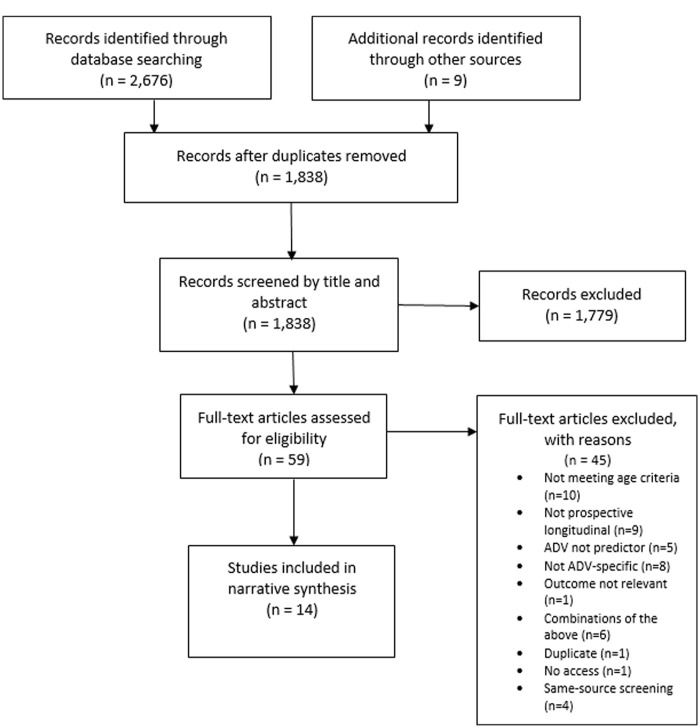
Flowchart of study selection.

The searches generated 2,676 records. Nine further references in the reference lists of similar reviews were considered for screening. After removing duplicates, 1,838 records were screened by title and abstract. Subsequently, the full text of 59 references that contained potentially relevant information were obtained and read. From this number, 41 publications were excluded. It was not possible to screen one of the publications from the search results due to it being inaccessible despite attempts to contact the author. In all, 18 publications met the inclusion criteria for the systematic review. However, four were discarded after applying the same-source screening criteria developed by the study team to avoid inflation of results. The criteria prioritized the publications with the highest methodological quality, specifically more thorough control of variables and bigger sample size (protocol indicating selection criteria for publications based on the same study is available upon request) when two or more publications were based on the same data and looking at the same outcomes. Publications excluded at this final stage were Cui et al. (2013), Exner-Cortens et al. (2017), Yohros et al. (2018), and Mumford, Liu et al. (2019). We ensured that none of the included same-source publications looked at the same outcomes. The final sample for the present study is 14 publications based on 12 longitudinal studies (see Figure 1).

The selection criteria were applied by two independent researchers (LCT and SL) using EndNote X9 software (The EndNote Team, 2013). A meeting took place at the end of each screening stage (i.e., title screening, abstract screening, and full-text reading) to compare the two independent lists of included and excluded references. There were reasonable levels of agreement between the reviewers (69.23%–87.50%) and all disagreements were resolved through thorough discussion.

### Data Extraction and Analysis

Data were extracted into an Excel spreadsheet by one researcher (LCT), with 100% of data being checked for accuracy by a second researcher (SL). A narrative approach was used to synthetsize findings. Furthermore, we employed simple descriptive statistics to summarize and present the proportion of associations between ADV victimization and outcomes, and to make comparisons according to the type of ADV experienced and the gender of the victim. Only evidence on outcome variables that were examined by at least two different publications was synthetized with the aim of avoiding re-stating information from the original article. For the analysis section on the comparison of findings by gender, only studies that separated their results for boys and girls were considered. It should be noted that some publications referred to gender and others to sex of participants, and many used terminologies on gender identity interchangeably. Following the American Psychiatric Association bias-free language guidelines, we keep the terminology on this aspect consistent across the manuscript (gender; girl(s)/boy(s)), but please note that this does not necessarily reflect the language used in the included publications.

It is also important to note that there is a lack of consensus in the field of intimate partner research regarding the definition of psychological violence, with theoretical debates in relation to the domains that should be included in the definition of this form of violence, as it is the case of controlling behaviors (Follingstad, 2009; Heise et al., 2019). Given the limited availability of longitudinal research with relevant information to address our research questions, we synthetized evidence on controlling behaviors within the broader framework of psychological ADV for the purposes of this study. However, we indicated when the publications in question were reporting on controlling behaviors specifically.

### Quality Assessment

The quality of the reviewed studies was assessed using the Mixed Methods Appraisal Tool (MMAT; Hong et al., 2018) by two reviewers (LCT and SL).

## Results

### Methodological Aspects of the Studies Reviewed

#### Study Design and Origin of Reviewed Publications

All included studies had prospective cohort designs and were based on quantitative data. In all, 12 were scientific publications available in peer-reviewed journals. The remaining were a final study report (Copp & Johnson, 2015) and a doctoral thesis (Pierce, 2017). All publications were conducted in the same high-income country (i.e., the United States) and published in English.

We included publications based on the same data source in two occasions: Exner-Cortens et al. (2013) and Pierce (2017) on the Add Health study, and Mumford, Taylor et al. (2019) and B. Taylor et al. (2017) on The National Survey of Teen Relationships and Intimate Violence (STRiV). All four were ultimately included after applying the developed same-source screening criteria and considering they provided data on different outcomes. For information on main characteristics of the publications included, see Table 1.

**Table 1.** Main Characteristics of Reviewed Studies.

Author and Year	Data Source	Sample Size	Number of Waves	Interval(s) Between Waves	Types of ADV	Dependent Variables
Choi et al. (2017)	Unnamed	583	4	1 year	Phy, Psy	Substance use, externalizing behaviors
Copp and Johnson (2015)	TARS	NP	5	w1–w2: 1–2 years w2–w3: 2–3 years w3–w4: 2 years approx. w4–w5: 5 years approx.	Phys	Internalizing symptoms, well-being, revictimization
Exner-Cortens et al. (2013)	Add Health	5,681	3	w1–w2: 1–2 years w2–w3: 5–6 years	Phy, Psy	Internalizing symptoms, well-being, substance use, externalizing behaviors, revictimization
Foshee et al. (2013)	Context Study	3,328	4	w1–w3: 6 months W3–w4: 1 year	Phy, Psy	Internalizing symptoms, substance use
Mulla et al. (2020)	Masked	1,752	2	2 months	Phys, Sex	Substance use
Mumford, Taylor et al. (2019)	STRiV	261	3	1 year	Phy, Psy*, Sex	Internalizing symptoms
Nahapetyan et al. (2014)	Healthy Teens Longitudinal Study	556	4	1 year	Phys	Internalizing symptoms
Pierce (2017)	Add Health	591	3	w1–w2: 5–6 years w2–w3: 5–7 years	Phy, Psy	Well-being
Reyes et al. (2017)	Unnamed	210	2	6 months	Phy, Psy, Sex	Internalizing symptoms
Reyes et al. (2018)	Unnamed	3,068	2	6 months	Phy, Psy*, Sex	Internalizing symptoms, substance use
Shorey et al. (2015)	Dating it Safe	882	2	1 year	Phy, Psy, Sex	Externalizing behaviors
Smith et al. (2003)	Unnamed	1,569	4 + 1 retrospective	1 year	Phys	Revictimization
B. Taylor et al. (2017)	STRiV	346	2	1 year	Phy, Psy*, Sex	Externalizing behaviors, revictimization
K. A. Taylor and Sullivan (2017)	MVPP	2,022	2	6 months	Phy, Psy	Substance use, revictimization

*Note.* ADV = adolescent dating violence; Add Health = The National Longitudinal Study of Adolescent to Adult Health; MVPP = Multisite Violence Prevention Project; NP = Not provided; STRiV = The National Survey of Teen Relationships and Intimate Violence; TARS = Toledo Adolescent Relationships Study.

* Mumford, Taylor et al. (2019) and B. Taylor et al. (2017) measure controlling behaviors. Reyes et al. (2018) measure verbal violence specifically.

Three publications used samples that had been originally recruited as part of randomized controlled trials (RCTs) for implementing violence prevention programs (Mulla et al., 2020; Reyes et al., 2017; K. A. Taylor & Sullivan, 2017). Mulla et al. (2020)’s sample was part of an ongoing RCT for a school-based sexual assault prevention program and Reyes et al. (2017) used data from a RCT of an ADV prevention program for Latino caregivers and youth. In K. A. Taylor and Sullivan (2017)’s study, none of the interventions specifically targeted dating violence victimization or outcome (i.e., substance use) prevention. Additional methodological details of the publications included (i.e., sampling strategy and mode of survey administration) are found in the Supplemental Materials.

#### Frequency and Severity Indicators

We identified a general lack of indicators of frequency and severity used in the analyses of the reviewed publications. Although half of the publications included frequency response scales for measuring ADV victimization, in most cases the responses were ultimately dichotomized to victimized/non-victimized categories. The publication by Reyes et al. (2018) was the only one that consistently reported results according to both severity (i.e., moderate, severe) and frequency (i.e., occasional, frequent) of victimization.

#### Perpetration and Poly-Victimization

Although the focus of the current study is victimization, we considered important to extract data on two variables that are relevant in the context of adolescent victimization: ADV perpetration and poly-victimization (Hamby et al., 2018; Spencer et al., 2020). First, it should be noted that more than half of the publications included (8/14) measured whether participants, in addition to being victimized, exerted any perpetrating behaviors toward their dating partners. Some publications reported results having measured victimization concurrently with perpetration (Choi et al., 2017; Reyes et al., 2017, 2018), specifically in groups for latent class analyses. The remaining publications that measured perpetration in addition to victimization reported results separately (Copp & Johnson, 2015; Nahapetyan et al., 2014; Shorey et al., 2015; K. A. Taylor & Sullivan, 2017; B. Taylor et al., 2017). Second, of the total 14 publications included, less than half explored other victimization experiences. These focused mainly on childhood abuse (Exner-Cortens et al., 2013; Pierce, 2017; Smith et al., 2003), sexual violence perpetrated by anyone (Exner-Cortens et al., 2013; Mulla et al., 2020; Smith et al., 2003), peer violence aggression and victimization (Reyes et al., 2018), parental physical abuse, and witnessing domestic violence (Smith et al., 2003). Multiple victimization experiences were measured with the purpose of either being controlled for in the analyses (Exner-Cortens et al., 2013; Pierce, 2017), explored as independent variables (Mulla et al., 2020; Smith et al., 2003), or included in groups for latent class analysis (Reyes et al., 2018).

### Quality Assessment

A quality assessment was conducted on the 14 publications using the MMAT (Hong et al., 2018; see Appendix 1 for detailed information). Overall, all the reviewed publications demonstrate an adequate methodological quality. Over half of the publications met all five MMAT quality criteria, which covered representativity of sample, appropriate use of instruments to measure variables, availability of complete outcome data, accounting for confounders in the design and analysis, and whether the exposure occurred among the participants as expected (Choi et al., 2017; Copp & Johnson, 2015; Exner-Cortens et al., 2013; Mulla et al., 2020; Mumford, Taylor et al., 2019; Nahapetyan et al., 2014; Pierce, 2017; Reyes et al., 2018; B. Taylor et al., 2017). It should be noted that K. A. Taylor and Sullivan (2017)’s sample was characterized by higher-than-average poverty rate and Reyes et al. (2017) had a small sample of Latino participants.

### Characteristics of the Samples Included

The total sample of this review is 19,997 (selecting the publication with the highest sample size in cases of same-source data and considering one publication did not report data on this). Across included publications, sample sizes ranged widely, from 210 to 5,681.

All publications measured dating violence during adolescence (10–19 years old). In terms of adolescence sub-periods based on previous literature (e.g., Mulwa et al., 2021; World Health Organization, 2010), two publications measured ADV during early adolescence (10–14 years old; K. A. Taylor & Sullivan, 2017; B. Taylor et al., 2017), four during middle adolescence (15–17 years old; Mulla et al., 2020; Mumford, Taylor et al., 2019; Pierce, 2017; Shorey et al., 2015), and one in late adolescence (18–19 years old; Smith et al., 2003). The remaining measured the predicting variable in multiple occasions coinciding with different adolescent stages.

Results show that there was a clear majority of White participants in 5/14 of publications included (Exner-Cortens et al., 2013 and Pierce, 2017 on the Add Health; Reyes et al., 2018; Smith et al., 2003; B. Taylor et al., 2017) and a relative ethnically diverse in 4/14 publications (Choi et al., 2017; Foshee et al., 2013; Nahapetyan et al., 2014; Shorey et al., 2015). One publication involved a majority of Black participants (K. A. Taylor & Sullivan, 2017) and one had a sample formed by participants with Latino heritages (Reyes et al., 2017). Three publications did not provide information on race or ethnicity of participants. Most publications classified gender in their samples binarily and had balanced distributions between boys and girls, with the exception of two publications that included girl participants exclusively (Choi et al., 2017; Smith et al., 2003). See Table 2 for sociodemographic information. All publications referred to heterosexual relationships. None of the studies captured at follow-up whether participants were in a relationship with the same partner that had perpetrated dating violence at baseline. Two studies collected data from rural areas (Foshee et al., 2013; Reyes et al., 2018).

**Table 2.** Sociodemographic Information of Samples Included.

Author and Year	Country	Gender	Mean Age When ADV	Ethnicity	Education (Baseline)
Choi et al. (2017)	USA	100% female	15.1 (0.78)	32% Hispanic, 30% White, 26% African American, 12% Other	9th–11th grades
Copp and Johnson (2015)	USA	NP	NP	NP	7th, 9th, and 11th grades
Exner-Cortens et al. (2013)	USA	47.7% male; 52.3% female	16.0 (0.10); range 12–18	69.3% White, non-Hispanic; 13.5% Black, non-Hispanic; 10.8% Hispanic; 6.4% Other	7th–12th grades
Foshee et al. (2013)	USA	49% male; 51% female	NP	50% Black, 43% White, 7% Other (Latino, Asian, American Indian, or mixed race)	8th–12th grades
Mulla et al. (2020)	USA	47.5% male; 52.5% female	15.38 (0.63)	NP	10th grade
Mumford, Taylor et al. (2019)	USA	52.9% male; 47.1% female	15.6; range 10–18	NP	NP
Nahapetyan et al. (2014)	USA	50.2 % male; 49.8% female	NP	47.5% White, 37.8% Black, 11.2% Latino	9th–12th grade
Pierce (2017)	USA	38.9% male; 61.1% female	16.06 (1.46); range 13–18	66.5% White, 21% African-American, 4.7% Other, 4.4% multiracial, 2.7% Asian, 0.5% American Indian	8th–12th grade
Reyes et al. (2017)	USA	42% male; 58% female	13.87 years; range 12–16	75% Mexican descent, 9% Central American, 6% South American, 2% Puerto Rican, 8% mixed/other heritage	NP
Reyes et al. (2018)	USA	54% male; 46% female	NP	58% White, 31% Black, 11% Other	8th–10th grade
Shorey et al. (2015)	USA	44.2% male; 55.8% female	15.02 (0.72) male; 15.01 (0.69) female	33.2% Caucasian, 31.5% African American, 35.3% Hispanic	9th–10th grade
Smith et al. (2003)	USA	100% female	Range 18–19	70.9% White, 25.3% Black, 3.8% Other	1st college year
B. Taylor et al. (2017)	USA	50.9% male; 49.1% female	NP	72.8% White, non-Hispanic	6th grade
K. A. Taylor & Sullivan (2017)	USA	57% male; 43% female	NP	55% Black, 17% Latino/a, 16% White, 9% as multiracial, 3% Other	NP

*Note.* ADV = adolescent dating violence; NP = not provided.

### Outcomes

Publications reviewed provided evidence on 11 different outcomes that were grouped in the categories of internalizing symptoms (depressive symptoms, anxiety symptoms, and suicidal ideation), externalizing behaviors (ADV perpetration, antisocial behaviors—including delinquency, and sexual risk behaviors), well-being (self-rated health), substance use (including alcohol, marijuana, and tobacco), and revictimization.

Overall, the 14 reviewed publications explored a total of 118 unique associations between distinct forms of ADV and the explored outcomes listed above. From these, 51 different associations were explored in samples or subsamples formed by girls, 42 in boys subsamples, and 23 in samples formed by boys and girls combined. In terms of forms of ADV, most associations involved physical (*n* = 33) and psychological (*n* = 41) ADV victimization. From the psychological subgroup, some associations were specifically on verbal abuse (*n* = 6) from the same publication (Reyes et al., 2018) and fewer on controlling behaviors by a partner (*n* = 3) based on two publications (Mumford, Taylor et al., 2019; B. Taylor et al. 2017). Other associations focused on combinations between several forms of ADV, such as physical and psychological (*n* = 21), integrated combinations of the three main forms of ADV (physical, psychological, sexual; *n* = 16), or psychological and sexual (*n* = 1). Less associations focused on the association between sexual ADV individually (*n* = 6) and the listed outcomes.

#### By Form of Violent Victimization

Most publications explored combinations of different forms of ADV victimization (Table 1). For most outcomes, the significance of the association with earlier ADV differed by the form of violence experienced. It should be noted psychological violence did not consistently predict the reviewed outcomes, regardless of gender, as there was roughly the same number of significant and non-significant associations across studies. Although supported by a lower number of studies, verbal abuse, as a specific form of psychological ADV, showed a tendency to be significantly associated with adverse outcomes, particularly in girls. In samples formed by boys and girls for which results were provided without a breakdown by gender, sexual, and psychological ADV led to more non-significant associations with adverse outcomes, than to significant associations. As in samples or subsamples formed by girls, physical ADV was linked to more significant associations, therefore, to a broader range of adverse outcomes.

#### By Gender of the Victim

Although with some differences, overall, the reviewed evidence suggests that the association between victimization and some outcomes (significant and non-significant) was similarly reported for boys and girls. These included depressive symptoms, self-rated health, ADV perpetration, sexual risk behaviors, and revictimization. Some publications reported outcome findings for boys and girls combined and, therefore, comparisons between these two gender groups could not be made (Mulla et al., 2020; Pierce, 2017; Shorey et al., 2015; K. A. Taylor & Sullivan, 2017; B. Taylor et al., 2017).

**Figure 2.**
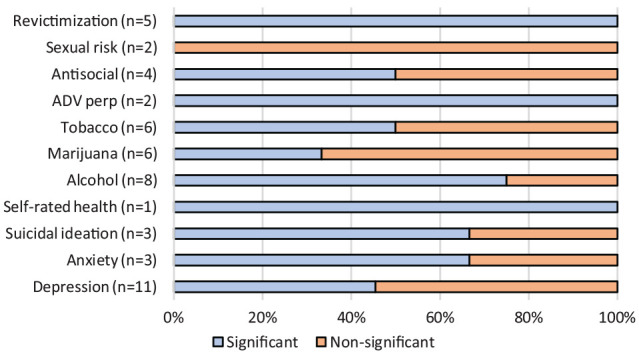
Proportion of significant and non-significant associations between ADV victimization and outcomes—girls. *Note.* ADV = adolescent dating violence; perp = perpetration.

**Figure 3.**
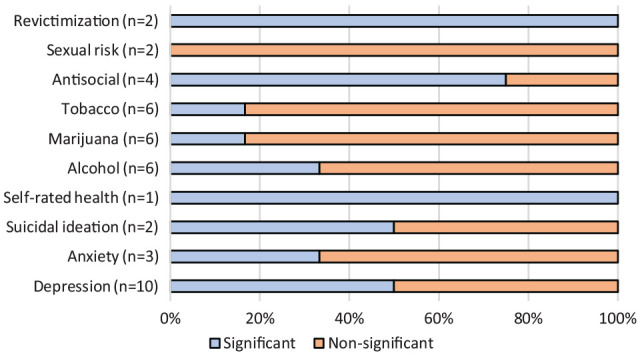
Proportion of significant and non-significant associations between adolescent dating violence victimization and outcomes—boys.

From the 51 associations explored in girls across the reviewed publications, around 59.0% suggested a significant link between the different forms of ADV victimization and the adverse outcomes (Figure 2). In particular, more studies found that girls self-reported higher substance use (including tobacco, marijuana, and alcohol), suicidal ideation, and anxiety symptoms than boys. In boys, the 42 explored associations had a slightly higher proportion of non-significant (59.5%) compared to significant (40.5%) longitudinal links between ADV and adverse outcomes (Figure 3). More studies suggested that boys who had experienced ADV victimization significantly self-reported antisocial behaviors, including delinquency, compared to girls.

Revictimization and poor self-rated health were significantly associated with earlier ADV, in boys and girls. In contrast, no studies that included samples or subsamples of boys and/or girls suggested that ADV may lead to sexual risk behaviors; this association was only found in a minority of publications with mixed-gender groups for analyses.

The publications that reported results concurrently for boys and girls had an important proportion of mixed findings, as results on substance use (alcohol and tobacco), ADV perpetration, and revictimization outcomes showed a similar number of significant and non-significant associations across reviewed publications.

## Discussion

We systematically selected and reviewed a total of 14 publications that met our eligibility criteria to gain a deeper understanding of the outcomes that follow experiences of ADV victimization. Special emphasis was given to the form of violence experienced and the gender of the victim with the aim of exploring any potential variations in the significance of these associations. We focused on studies with prospective cohort designs to substantiate the order of variables and establish a temporal relationship between ADV victimization and outcomes, a crucial condition for suggesting causality.

Overall, the most explored forms of ADV in the reviewed studies were physical and psychological violence. Sexual victimization received limited attention compared to the other forms of ADV, and was only explored individually in studies that did not report results separately by gender, missing the opportunity to take into account aspects of gender that could be linked to this form of ADV. This is a relevant finding considering that adolescents are more likely to be sexually victimized by a partner (42%) than by another well-known person to the victim (39%), stranger (21%), or family member (9%) (Averdijk et al., 2011). The predominance of the physical violence form is common in victimization studies, as it is also the lack of research on sexual violence victimization (e.g., Reyes et al., 2016; Rubio-Garay et al., 2017). The fact that sexual violence is often overlooked in ADV research, especially in adolescent samples, could be related to issues with compulsory reporting to the authorities in cases of abuse, or even difficulties by the schools agreeing to ask minors about sexuality. As Jackson (1999) already claimed over 20 years ago, the unbalanced proportion of studies on physical versus psychological and sexual violence limits knowledge on the field by not providing an integrated picture of the dating violence phenomenon.

Our findings suggest that experiencing ADV is longitudinally associated with many adverse outcomes, including some higher internalizing symptoms and externalizing behaviors, poorer well-being, increased substance use, and increased revictimization. However, this review shows that associations are not always consistent across studies when we take into account the form of ADV experienced and the gender of the victim. This may hamper generalization of results in instances where these variables are not considered. This could help explain inconsistency in findings in ADV research. Our study shows that depressive symptoms revealed one of the highest rates of discrepancies, whereas revictimization was the most consistent and well-supported outcome to be associated with earlier ADV. There are two important issues that deserve highlighting in this context. First, our analysis of study characteristics underscores the significant diversity of the methodological aspects considered across the publications, such as sampling strategies and mode of survey administration employed, which has been linked to influence reports of victimization (Ocampo et al., 2007; Rathod et al., 2011). Second, most of the outcomes were not examined across all forms of ADV and the number of studies supporting each association was limited. In fact, the total sample of publications in our current review reflects the scarcity of longitudinal research on the potential outcomes of ADV. It is important to note that while some of the outcomes did not consistently show significant associations with earlier ADV in our review, they have been supported in previous cross-sectional research, as evidenced in the review by Taquette and Monteiro (2019).

In addition, we found that longitudinal associations between ADV and the explored outcomes often differ by gender of victims, with some outcomes suggesting gender-dependent associations while not necessarily adhering to patterns of female internalizing and male externalizing, as other studies with younger samples previously suggested (Dulmus & Hilarski, 2006; Maschi et al., 2008). Overall, girls were more prone to experience adverse outcomes after ADV victimization, compared to boys, which is in line with previous research (Hamby & Turner, 2013; Hébert et al., 2017; Théorêt et al., 2021). This could be related to other components that the studies reviewed did not consistently capture, such as increased severity and frequency of violence experienced or greater fear perceived by girls, which have been previously associated with poorer symptoms in intimate partner violence (Hegarty et al., 2013; Jaquier & Sullivan, 2014; Sabri, 2012). In addition, very few studies included indicators of frequency. This is a relevant variable to consider given that measuring degrees of severity separately prevents overestimation of prevalence rates (Smith et al., 2003). Regarding other potential factors that may allow to better understand the outcomes of ADV victimization, in this study no clear patterns involving both gender and adolescence stage (i.e., early, middle, late) were found in relation to the outcomes. However, it should be noted that there was no balanced data on adolescence stages and there was a clear lack of ADV measured in late adolescence. In terms of race and ethnic groups of sample participants, under half of publications included diverse samples. While there were a few studies with a greater proportion of more underrepresented groups in research, it should be noted that all publications were based in the context of a high-income country, lacking representativity of low- and middle-income countries.

It is crucial to acknowledge that none of the publications included in our review provided evidence on ADV experiences in non-heterosexual relationships. This is significant considering that a substantial proportion of the general population identifies as non-heterosexual (e.g., Green et al., 2019) and that research has shown higher rates of ADV victimization and perpetration among LGBTQ+ populations (Zweig et al., 2013). Therefore, there is a pressing need for more research that includes samples with diverse sexual orientations and gender identities to better understand the unique vulnerabilities of youth to ADV, as well as the potential consequences of such experiences (Zweig et al., 2013).

There was a notable lack of evidence on multiple victimization experiences across studies, which hinders the understanding of the cumulative burden of victimization and its potential influence on the outcomes explored in this review. According to the poly-victimization framework, many youths experience inter-connected victimization in multiple settings and by multiple perpetrators (Hamby et al., 2018). Therefore, it is crucial to consider the possibility of overlapping victimization experiences to better comprehend the complex dynamics of ADV outcomes. Furthermore, it is important to acknowledge that the diverse symptomatology observed in victims of dating violence may also be influenced by other factors, such as socioeconomic and sociocultural factors, as it has been suggested in previous research on women victims of violence (Briere & Jordan, 2004; Table 3).

**Table 3.** Summary of Critical Findings.

Critical Findings
There is limited longitudinal research examining the outcomes of ADV victimization, which hinders associations suggesting causality
The significance of the longitudinal association between ADV victimization and adverse outcomes often varies based on the gender of the victim and the specific form of ADV experienced
While physical and psychological victimization in ADV receive significant attention, sexual violence is not explored to the same extent in this context, creating an imbalance in research and understanding of the different forms of ADV
There is a general lack of severity and frequency indicators in this research area
Studies on the outcomes of ADV victimization employ diverse method strategies, which may help explain variability of results across research
Victimization experiences that may precede or co-occur with ADV are not consistently explored in research
There is a clear lack of diversity in the samples used to better understand the impact of ADV victimization, especially in terms of ethnicity, sex identity, and sexual orientation of participants, and world region (i.e., low- and middle-income countries) where the studies are conducted

*Note.* ADV = adolescent dating violence.

This study has some strengths that deserve recognition. First, to our knowledge, this is the first comprehensive systematic review that specifically examined longitudinal research on the association between ADV victimization and adverse outcomes. Second, our review is based on a comprehensive and systematic search strategy, ensuring a rigorous and thorough approach to identifying relevant studies. Third, our study analyzes in detail how different forms of violence and the gender of the victims may influence the outcomes following ADV, offering valuable insights into the nuanced complexities of ADV research.

However, several limitations of this review should also be acknowledged. First, the generalizability of the results may be limited, as associations between dating violence and outcomes were often supported by a low number of studies, and samples included in the studies were not geographically diverse. Therefore, caution should be used when applying the findings to different populations or settings. Second, it should be noted that the reviewed studies only reported victimization experiences in heterosexual couples, which may not fully capture the experiences of individuals in non-heterosexual relationships. Lastly, it is important to highlight that this review primarily focuses on ADV victimization, and further analysis on how perpetration and multiple types of victimization influence outcomes could provide additional insights into the complex dynamics of ADV outcomes.

## Conclusion

Our findings suggest that experiencing ADV is longitudinally associated with many adverse outcomes, including higher internalizing symptoms and externalizing behaviors, poorer well-being, increased substance use, and increased revictimization. However, the review highlights the complex and diverse patterns of associations between ADV victimization and various adverse outcomes, as these associations appear to be influenced by multiple factors, including the form of violence experienced and the gender of the victim, possibly in addition to other factors, such as the various methodological research aspects across studies on the subject. Due to the relative inconsistencies of ﬁndings and the limited number of studies reporting results on each outcome, no ﬁrm conclusions can be drawn.

### Implications for Research, Policy, and Practice

- *Enhanced longitudinal data*: There is a clear need for more robust longitudinal data that explores the potential outcomes of ADV victimization. This should include analyses that differentiate between the various forms of violence experienced, as well as taking into account the gender of the victims. This will provide a more comprehensive understanding of the effects of ADV victimization.
- *Inclusion of various forms of dating ADV victimization:* New studies should strive to capture the unique impact of different forms of dating ADV victimization and in a more balanced way, including emerging types of victimization prevalent in the adolescent population. This will help broaden the evidence base and provide a more nuanced understanding of the dynamics and consequences of dating victimization.
- *Consistent consideration of relevant indicators:* Future research on ADV victimization should consistently incorporate indicators of severity and frequency. This will enable a more accurate and comprehensive assessment of the extent and impact of victimization, providing valuable insights into the dynamics and consequences of ADV.
- *Comprehensive assessment of victimization experiences:* It is important to assess other victimization experiences that may precede or co-occur with dating violence. This holistic approach to assessment will provide a more comprehensive understanding of the overall impact of victimization.
- *Inclusive research:* It is imperative to explore the potential outcomes of dating violence in diverse populations, such as low- and middle-income countries and LGTBQ+ populations. Understanding the unique vulnerabilities and experienced outcomes of ADV in diverse populations is crucial for developing effective interventions and policies.
- *Holistic approach to prevention and intervention:* In addition to primary prevention policies, policymakers should also consider tertiary prevention strategies that aim to mitigate the effects of dating violence when it has already occurred. This includes mechanisms for appropriate treatment, monitoring of adolescent victims to prevent revictimization, and addressing the complex and varied symptomatology that follows victimization. A comprehensive approach that encompasses prevention and intervention strategies is crucial in addressing the multifaceted impact of ADV.

## Supplemental Material

sj-docx-1-tva-10.1177_15248380231174504 – Supplemental material for Longitudinal Associations Between Adolescent Dating Violence Victimization and Adverse Outcomes: A Systematic Review Supplemental material, sj-docx-1-tva-10.1177_15248380231174504 for Longitudinal Associations Between Adolescent Dating Violence Victimization and Adverse Outcomes: A Systematic Review by Laura Campo-Tena, Simon R. Larmour, Noemí Pereda and Manuel P. Eisner in Trauma, Violence, & Abuse

sj-docx-2-tva-10.1177_15248380231174504 – Supplemental material for Longitudinal Associations Between Adolescent Dating Violence Victimization and Adverse Outcomes: A Systematic Review Supplemental material, sj-docx-2-tva-10.1177_15248380231174504 for Longitudinal Associations Between Adolescent Dating Violence Victimization and Adverse Outcomes: A Systematic Review by Laura Campo-Tena, Simon R. Larmour, Noemí Pereda and Manuel P. Eisner in Trauma, Violence, & Abuse

sj-docx-3-tva-10.1177_15248380231174504 – Supplemental material for Longitudinal Associations Between Adolescent Dating Violence Victimization and Adverse Outcomes: A Systematic Review Supplemental material, sj-docx-3-tva-10.1177_15248380231174504 for Longitudinal Associations Between Adolescent Dating Violence Victimization and Adverse Outcomes: A Systematic Review by Laura Campo-Tena, Simon R. Larmour, Noemí Pereda and Manuel P. Eisner in Trauma, Violence, & Abuse

sj-docx-4-tva-10.1177_15248380231174504 – Supplemental material for Longitudinal Associations Between Adolescent Dating Violence Victimization and Adverse Outcomes: A Systematic Review Supplemental material, sj-docx-4-tva-10.1177_15248380231174504 for Longitudinal Associations Between Adolescent Dating Violence Victimization and Adverse Outcomes: A Systematic Review by Laura Campo-Tena, Simon R. Larmour, Noemí Pereda and Manuel P. Eisner in Trauma, Violence, & Abuse

## References

[bibr1-15248380231174504] Ackard D. M. Eisenberg M. E. Neumark-Sztainer D. (2007). Long-term impact of adolescent dating violence on the behavioral and psychological health of male and female youth. The Journal of Pediatrics, 151(5), 476–481. 10.1016/j.jpeds.2007.04.034 17961688

[bibr2-15248380231174504] Alleyne-Green B. Grinnell-Davis C. Clark T. T. Quinn C. R. Cryer-Coupet Q. R. (2016). Father involvement, dating violence, and sexual risk behaviors among a national sample of adolescent females. Journal of Interpersonal Violence, 31(5), 810–830. 10.1177/0886260514556762 25475102 PMC5007216

[bibr3-15248380231174504] Averdijk M. Mueller-Johnson K. Eisner M. (2011). Sexual victimization of children and adolescents in Switzerland (Final report for the UBS Optimus Foundation). UBS Optimus Foundation.

[bibr4-15248380231174504] Baker C. K. (2016). Dating violence and substance use: Exploring the context of adolescent relationships. Journal of Interpersonal Violence, 31(5), 900–919. 10.1177/0886260514556768 25395224

[bibr5-15248380231174504] Barnes J. E. Noll J. G. Putnam F. W. Trickett P. K. (2009). Sexual and physical revictimization among victims of severe childhood sexual abuse. Child Abuse & Neglect, 33(7), 412–420. 10.1016/j.chiabu.2008.09.013 19596434 PMC2723796

[bibr6-15248380231174504] Barros C. Schraiber L. B. França-Junior I. (2011). Association between intimate partner violence against women and HIV infection. Revista de Saude Publica, 45, 365–372. 10.1590/s0034-89102011005000008 21344126

[bibr7-15248380231174504] Briere J. Jordan C. E. (2004). Violence against women: Outcome complexity and implications for assessment and treatment. Journal of Interpersonal Violence, 19(11), 1252–1276. 10.1177/0886260504269682 15534329

[bibr8-15248380231174504] Burton C. W. Halpern-Felsher B. Rehm R. S. Rankin S. H. Humphreys J. C. (2016). Depression and self-rated health among rural women who experienced adolescent dating abuse: A mixed methods study. Journal of Interpersonal Violence, 31(5), 920–941. 10.1177/0886260514556766 25392389

[bibr9-15248380231174504] Centers for Disease Control & Prevention. (2017). Injury prevention and control division of violence prevention understanding teen dating violence.

[bibr10-15248380231174504] Choi H. J. Elmquist J. Shorey R. C. Rothman E. F. Stuart G. L. Temple J. R. (2017). Stability of alcohol use and teen dating violence for female youth: A latent transition analysis. Drug and Alcohol Review, 36(1), 80–87. 10.1111/dar.12462 28109181 PMC5280082

[bibr11-15248380231174504] Coker A. L. McKeown R. E. Sanderson M. Davis K. E. Valois R. F. Huebner E. S. (2000). Severe dating violence and quality of life among South Carolina high school students. American Journal of Preventive Medicine, 19(4), 220–227. 10.1016/S0749-3797(00)00227-0 11064224

[bibr12-15248380231174504] Copp J. E. Giordano P. C. Longmore M. A. Manning W. D. (2016). Dating violence and physical health: A longitudinal lens on the significance of relationship dynamics and anti-social lifestyle characteristics. Criminal Behaviour and Mental Health, 26(4), 251–262. 10.1002/cbm.2016 27709746 PMC7946329

[bibr13-15248380231174504] Copp J. E. Johnson W. L. (2015). Patterns, precursors, and consequences of teen dating violence: Analyzing gendered and generic pathway (Final Report to the National Institute of Justice).

[bibr14-15248380231174504] Cui M. Ueno K. Gordon M. Fincham F. D. (2013). The continuation of intimate partner violence from adolescence to young adulthood. Journal of Marriage and Family, 75(2), 300–313. 10.1111/jomf.12016 23687386 PMC3653598

[bibr15-15248380231174504] Dulmus C. N. Hilarski C. (2006). Significance of gender and age in African American children’s response to parental victimization. Health & Social Work, 31(3), 181–188. 10.1093/hsw/31.3.181 16955656

[bibr16-15248380231174504] Epstein-Ngo Q. M. Cunningham R. M. Whiteside L. K. Chermack S. T. Booth B. M. Zimmerman M. A. Walton M. A. (2013). A daily calendar analysis of substance use and dating violence among high risk urban youth. Drug and Alcohol Dependence, 130(1–3), 194–200. 10.1016/j.drugalcdep.2012.11.006 23219602 PMC3622164

[bibr17-15248380231174504] Eshelman L. Levendosky A. A. (2012). Dating violence: Mental health consequences based on type of abuse. Violence and Victims, 27(2), 215–228. 10.1891/0886-6708.27.2.215 22594217

[bibr18-15248380231174504] Exner-Cortens D. Eckenrode J. Bunge J. Rothman E. (2017). Revictimization after adolescent dating violence in a matched, national sample of youth. Journal of Adolescent Health, 60(2), 176–183. 10.1016/j.jadohealth.2016.09.015 28109451

[bibr19-15248380231174504] Exner-Cortens D. Eckenrode J. Rothman E. (2013). Longitudinal associations between teen dating violence victimization and adverse health outcomes. Pediatrics, 131(1), 71–78. 10.1542/peds.2012-1029 23230075 PMC3529947

[bibr20-15248380231174504] Fix R. L. Nava N. Rodriguez R. (2021). Disparities in adolescent dating violence and associated internalizing and externalizing mental health symptoms by gender, race/ethnicity, and sexual orientation. Journal of Interpersonal Violence, 37(17–18), NP15130–NP15152. 10.1177/0886260521997944 33678044

[bibr21-15248380231174504] Follingstad D. R. (2009). The impact of psychological aggression on women’s mental health and behavior—the status of the field. Trauma, Violence, Abuse, 10(3), 271–89. 10.1177/1524838009334453 19460760

[bibr22-15248380231174504] Foshee V. A. Reyes H. L. M. Gottfredson N. C. Chang L.-Y. Ennett S. T. (2013). A longitudinal examination of psychological, behavioral, academic, and relationship consequences of dating abuse victimization among a primarily rural sample of adolescents. Journal of Adolescent Health, 53(6), 723–729. 10.1016/j.jadohealth.2013.06.016 PMC3838452 23910572

[bibr23-15248380231174504] Green A. E. Price-Feeney M. Dorison S. (2019). National estimate of LGBTQ youth seriously considering suicide. The Trevor Project. https://www.thetrevorproject.org/wp-content/uploads/2019/06/Estimating-Number-of-LGBTQ-Youth-Who-Consider-Suicide-In-the-Past-Year-Final.pdf

[bibr24-15248380231174504] Hamby S. Taylor E. Jones L. Mitchell K. J. Turner H. A. Newlin C. (2018). From poly-victimization to poly-strengths: Understanding the web of violence can transform research on youth violence and illuminate the path to prevention and resilience. Journal of Interpersonal Violence, 33(5), 719–739. 10.1177/0886260517744847 29411696

[bibr25-15248380231174504] Hamby S. Turner H. (2013). Measuring teen dating violence in males and females: Insights from the national survey of children’s exposure to violence. Psychology of Violence, 3(4), 323. 10.1037/a0029706

[bibr26-15248380231174504] Haynie D. L. Farhat T. Brooks-Russell A. Wang J. Barbieri B. Iannotti R. J. (2013). Dating violence perpetration and victimization among US adolescents: Prevalence, patterns, and associations with health complaints and substance use. Journal of Adolescent Health, 53(2), 194–201. 10.1016/j.jadohealth.2013.02.008 PMC3725188 23664626

[bibr27-15248380231174504] Hébert M. Blais M. Lavoie F. (2017). Prevalence of teen dating victimization among a representative sample of high school students in Quebec. International Journal of Clinical and Health Psychology, 17(3), 225–233. 10.1016/j.ijchp.2017.06.001 29308070 PMC5756072

[bibr28-15248380231174504] Hegarty K. L. O’Doherty L. J. Chondros P. Valpied J. Taft A. J. Astbury J. Brown S. J. Gold L. Taket A. Feder G. S. Gunn J. M. (2013). Effect of type and severity of intimate partner violence on women’s health and service use: Findings from a primary care trial of women afraid of their partners. Journal of Interpersonal Violence, 28(2), 273–94. 10.1177/0886260512454722 22929341

[bibr29-15248380231174504] Heise L. Pallitto C. García-Moreno C. Clark C. J. (2019). Measuring psychological abuse by intimate partners: Constructing a cross-cultural indicator for the sustainable development goals. SSM – Population Health, 9, 100377. 10.1016/j.ssmph.2019.100377 31993478 PMC6978474

[bibr30-15248380231174504] Higgins J. P. Thomas J. Chandler J. Cumpston M. Li T. Page M. J. Welch V. A. (2020). Cochrane handbook for systematic reviews of interventions (1119536618). https://training.cochrane.org/handbook/current 10.1002/14651858.ED000142 PMC10284251 31643080

[bibr31-15248380231174504] Holmes K. Sher L. (2013). Dating violence and suicidal behavior in adolescents. International Journal of Adolescent Medicine and Health, 25(3), 257–261. 10.1515/ijamh-2013-0059 24006321

[bibr32-15248380231174504] Hong Q. N. Pluye P. Fàbregues S. Bartlett G. Boardman F. Cargo M. Dagenais P. Gagnon M-P. Griffithsc F. Nicolau B. O’Cathain A. Rousseau M-C. Vedel I. (2018). Mixed Methods Appraisal Tool (MMAT), version 2018. Registration of Copyright (#1148552), Canadian Intellectual Property Office, Industry of Canada.

[bibr33-15248380231174504] Jackson S. M. (1999). Issues in the dating violence research: A review of the literature. Aggression and Violent Behavior, 4(2), 233–247. 10.1016/S1359-1789(97)00049-9

[bibr34-15248380231174504] Jaquier V. Sullivan T. P. (2014). Fear of past abusive partner(s) impacts current posttraumatic stress among women experiencing partner violence. Violence Against Women, 20(2), 208–27. 10.1177/1077801214525802 24590514 PMC3999088

[bibr35-15248380231174504] Leadbeater B. Connolly J. Temple J. R. (2018). Changing your status in a changing world: It is complicated! A developmental systems framework for understanding dating violence in adolescents and young adults. In Wolfe D. A. Temple J. R. (Eds.), Adolescent dating violence: Theory, research, and prevention (pp. 3–23). Academic Press.

[bibr36-15248380231174504] Leen E. Sorbring E. Mawer M. Holdsworth E. Helsing B. Bowen E. (2013). Prevalence, dynamic risk factors and the efficacy of primary interventions for adolescent dating violence: An international review. Aggression and Violent Behavior, 18(1), 159–174. 10.1016/j.avb.2012.11.015

[bibr37-15248380231174504] Lormand D. K. Markham C. M. Peskin M. F. Byrd T. L. Addy R. C. Baumler E. Tortolero S. R. (2013). Dating violence among urban, minority, middle school youth and associated sexual risk behaviors and substance use. Journal of School Health, 83(6), 415–421. 10.1111/josh.12045 23586886 PMC4372798

[bibr38-15248380231174504] Martz D. M. Jameson J. P. Page A. D. (2016). Psychological health and academic success in rural Appalachian adolescents exposed to physical and sexual interpersonal violence. American Journal of Orthopsychiatry, 86(5), 594–601. 10.1037/ort0000174 27148751

[bibr39-15248380231174504] Maschi T. Morgen K. Bradley C. Hatcher S. S. (2008). Exploring gender differences on internalizing and externalizing behavior among maltreated youth: Implications for social work action. Child and Adolescent Social Work Journal, 25(6), 531–547. 10.1007/s10560-008-0139-8

[bibr40-15248380231174504] Miranda-Mendizabal A. Castellví P. Parés-Badell O. Alayo I. Almenara J. Alonso I. Blasco M. J. Cebrià A. Gabilondo A. Gili M. Lagares C. Piqueras J. A. Rodríguez-Jiménez T. Rodríguez-Marín J. Roca M. Soto-Sanz V. Vilagut G. Alonso J. (2019). Gender differences in suicidal behavior in adolescents and young adults: Systematic review and meta-analysis of longitudinal studies. International Journal of Public Health, 64(2), 265–283. 10.1007/s00038-018-1196-1 30635683 PMC6439147

[bibr41-15248380231174504] Mulla M. M. Bogen K. W. Orchowski L. M. (2020). The mediating role of school connectedness in the associations between dating and sexual violence victimization and substance use among high school students. Preventive Medicine, 139, 106197. 10.1016/j.ypmed.2020.106197 32652131

[bibr42-15248380231174504] Mulwa S. Osindo J. Wambiya E. O. Gourlay A. Maina B. W. Orindi B. O. Floyd S. Ziraba A. Birdthistle I. (2021). Reaching early adolescents with a complex intervention for HIV prevention: Findings from a cohort study to evaluate DREAMS in two informal settlements in Nairobi, Kenya. BMC Public Health, 21(1), 1–13. 10.1186/s12889-021-11017-y 34112119 PMC8194171

[bibr43-15248380231174504] Mumford E. A. Liu W. Taylor B. G. (2019). Youth and young adult dating relationship dynamics and subsequent abusive outcomes. Journal of Adolescence, 72, 112–123. 10.1016/j.adolescence.2019.02.013 30878691

[bibr44-15248380231174504] Mumford E. A. Taylor B. G. Liu W. Giordano P. C. (2019). Dating relationship dynamics, mental health, and dating victimization: A longitudinal path analysis. Journal of Research on Adolescence, 29(3), 777–791. 10.1111/jora.12415 29911354 PMC6941487

[bibr45-15248380231174504] Murray J. Farrington D. P. Eisner M. P. (2009). Drawing conclusions about causes from systematic reviews of risk factors: The Cambridge Quality Checklists. Journal of Experimental Criminology, 5(1), 1–23. 10.1007/s11292-008-9066-0

[bibr46-15248380231174504] Nahapetyan L. Orpinas P. Song X. Holland K. (2014). Longitudinal association of suicidal ideation and physical dating violence among high school students. Journal of Youth and Adolescence, 43(4), 629–640. 10.1007/s10964-013-0006-6 23996215

[bibr47-15248380231174504] Ocampo B. W. Shelley G. A. Jaycox L. H. (2007). Latino teens talk about help seeking and help giving in relation to dating violence. Violence Against Women, 13(2), 172–189. 10.1177/1077801206296982 17251504

[bibr48-15248380231174504] Page M. J. McKenzie J. E. Bossuyt P. M. Boutron I. Hoffmann T. C. Mulrow C. D. Brennan S. E. Chou R. Glanville J. Grimshaw J. M. Hróbjartsson A. Lalu M.M Li T. Loder E. W. Mayo-Wilson E. McDonald S. McGuinness L. A. Stewart L. A. Thomas J. , . . . Moher D. (2021). The PRISMA 2020 statement: An updated guideline for reporting systematic reviews. BMJ, 372, n71. 10.1136/bmj.n71 PMC8005924 33782057

[bibr49-15248380231174504] Pierce J. M. (2017). Longitudinal effects of adolescent dating violence victimization: Social, psychological, and physical health consequences in adulthood [Doctoral dissertation, Wayne State University], p. 1857. https://digitalcommons.wayne.edu/oa_dissertations/1857

[bibr50-15248380231174504] Rathod S. D. Minnis A. M. Subbiah K. Krishnan S. (2011). ACASI and face-to-face interviews yield inconsistent estimates of domestic violence among women in India: The Samata Health Study 2005–2009. Journal of Interpersonal Violence, 26(12), 2437–2456. 10.1177/0886260510385125 21282116 PMC3126890

[bibr51-15248380231174504] Reyes H. L. M. Foshee V. A. Chen M. S. Ennett S. T. (2017). Patterns of dating violence victimization and perpetration among Latino youth. Journal of Youth and Adolescence, 46(8), 1727–1742. 10.1007/s10964-016-0621-0 28005228 PMC5481538

[bibr52-15248380231174504] Reyes H. L. M. Foshee V. A. Chen M. S. Gottfredson N. C. Ennett S. T. (2018). Consequences of involvement in distinct patterns of adolescent peer and dating violence. Journal of Youth and Adolescence, 47(11), 2371–2383. 10.1007/s10964-018-0902-x 30043190 PMC6360938

[bibr53-15248380231174504] Reyes H. L. M. Foshee V. A. Niolon P. H. Reidy D. E. Hall J. E. (2016). Gender role attitudes and male adolescent dating violence perpetration: Normative beliefs as moderators. Journal of Youth and Adolescence, 45(2), 350–360. https://doi.org/0.1007/s10964-015-0278-0 25831994 10.1007/s10964-015-0278-0 PMC4592366

[bibr54-15248380231174504] Rubio-Garay F. López-González M. A. Carrasco M. Á. Amor P. J. (2017). The prevalence of dating violence: A systematic review. Papeles del Psicólogo, 38(2), 135–147. 10.23923/pap.psicol2017.2831

[bibr55-15248380231174504] Sabri B. (2012). Severity of victimization and co-occurring mental health disorders among substance using adolescents. Child Youth Care Forum, 41(1), 37–55. 10.1007/s10566-011-9151-9 23204820 PMC3507377

[bibr56-15248380231174504] Shorey R. C. Fite P. J. Choi H. Cohen J. R. Stuart G. L. Temple J. R. (2015). Dating violence and substance use as longitudinal predictors of adolescents’ risky sexual behavior. Prevention Science, 16(6), 853–861. https://doi.org/0.1007/s11121-015-0556-9 25797949 10.1007/s11121-015-0556-9 PMC4499016

[bibr57-15248380231174504] Shorey R. C. Sherman A. E. Kivisto A. J. Elkins S. R. Rhatigan D. L. Moore T. M. (2011). Gender differences in depression and anxiety among victims of intimate partner violence: The moderating effect of shame proneness. Journal of Interpersonal Violence, 26(9), 1834–1850. 10.1177/0886260510372949 20587460

[bibr58-15248380231174504] Smith P. H. White J. W. Holland L. J. (2003). A longitudinal perspective on dating violence among adolescent and college-age women. American Journal of Public Health, 93(7), 1104–1109. 10.2105/ajph.93.7.1104 12835193 PMC1447917

[bibr59-15248380231174504] Spencer C. M. Anders K. M. Toews M. L. Emanuels S. K. (2020). Risk markers for physical teen dating violence victimization in the United States: A meta-analysis. Journal of Youth and Adolescence, 49(3), 575–589. 10.1007/s10964-020-01194-1 31974737

[bibr60-15248380231174504] Taquette S. R. Monteiro D. L. M. (2019). Causes and consequences of adolescent dating violence: a systematic review. Journal of Injury and Violence Research, 11(2), 137. 10.5249/jivr.v11i2.1061 31263089 PMC6646825

[bibr61-15248380231174504] Taylor B. Joseph H. Mumford E. (2017). Romantic relationship characteristics and adolescent relationship abuse in a probability-based sample of youth. Journal of Interpersonal Violence, 36(1–2), 722–750. 10.1177/0886260517730566 29294910

[bibr62-15248380231174504] Taylor K. A. Sullivan T. N. (2017). Bidirectional relations between dating violence victimization and substance use in a diverse sample of early adolescents. Journal of Interpersonal Violence, 36(1–2), 862–891. 10.1177/0886260517731312 29294917

[bibr63-15248380231174504] Temple J. R. Freeman D. H. Jr. (2011). Dating violence and substance use among ethnically diverse adolescents. Journal of Interpersonal Violence, 26(4), 701–718. 10.1177/0886260510365858 20587475

[bibr64-15248380231174504] The EndNote Team. (2013). EndNote (Version EndNote X9) [64 bit]. Clarivate.

[bibr65-15248380231174504] Théorêt V. Hébert M. Fernet M. Blais M. (2021). Gender-specific patterns of teen dating violence in heterosexual relationships and their associations with attachment insecurities and emotion dysregulation. Journal of Youth and Adolescence, 50(2), 246–259. 10.1007/s10964-020-01328-5 33123947

[bibr66-15248380231174504] Tomaszewska P. Schuster I. (2021). Prevalence of teen dating violence in Europe: A systematic review of studies since 2010. New Directions for Child and Adolescent Development, 2021(178), 11–37. 10.1002/cad.20437 34724332

[bibr67-15248380231174504] Turner S. Norman E. Zunz S. (1995). Enhancing resiliency in girls and boys: A case for gender specific adolescent prevention programming. Journal of Primary Prevention, 16(1), 25–38. 10.1007/BF02407231 24254657

[bibr68-15248380231174504] Werner E. E. Smith R. S. (1982). Vulnerable but invincible: A longitudinal study of children and youth. Adams Bannister Cox Pubs.

[bibr69-15248380231174504] Werner E. E. Smith R. S. (1992). Overcoming the odds: High risk children from birth to adulthood. Cornell University Press.

[bibr70-15248380231174504] Wincentak K. Connolly J. Card N. (2017). Teen dating violence: A meta-analytic review of prevalence rates. Psychology of Violence, 7(2), 224–241. 10.1037/a0040194

[bibr71-15248380231174504] World Health Organization. (2010). Participant manual: IMAI one-day orientation on adolescents living with HIV. https://apps.who.int/iris/bitstream/handle/10665/44258/9789241598972_eng.pdf

[bibr72-15248380231174504] Yohros A. Ford J. Hinojosa M. S. (2018). Dating violence victimization and substance use: The role of a serotonin transporter gene polymorphism (5HTTLPR). Drug and Alcohol Dependence, 189, 178–186. 10.1016/j.drugalcdep.2018.05.003 30049530

[bibr73-15248380231174504] Zweig J. M. Dank M. Lachman P. Yahner J. (2013). Technology, teen dating violence and abuse, and bullying. Urban Institute.

