# Supplementary material for: Longitudinal Associations Between Adolescent Dating Violence Victimization and Adverse Outcomes: A Systematic Review
Source: Trauma Violence Abuse. 2023 May 25;25(2):1265–77. doi: 10.1177/15248380231174504 (PMC10913345; doi:10.1177/15248380231174504)
Supplement: sj-docx-1-tva-10.1177_15248380231174504 – Supplemental material for Longitudinal Associations Between Adolescent Dating Violence Victimization and Adverse Outcomes: A Systematic Review [file sj-docx-1-tva-10.1177_15248380231174504.docx]

**Appendix 1**.

## *Results of Quality Assessment (MMAT; Hong et al., 2018)*

| **Methodological quality criteria** | **Responses** | | | |
| --- | --- | --- | --- | --- |
|  | Yes | No | Can’t tell | Comments |
| S1. Are there clear research questions? | 1, 2, 3, 4, 5, 6, 7, 8, 9, 10, 11, 12, 13, 14 |  |  |  |
| S2. Do the collected data allow to address the research questions? | 1, 2, 3, 4, 5, 6, 7, 8, 9, 10, 11, 12, 13, 14 |  |  |  |
| *Further appraisal may not be feasible or appropriate when the answer is ‘No’ or ‘Can’t tell’ to one or both screening questions.* | | | | |
| 3.1. Are the participants representative of the target population? | 1, 2, 3, 4, 5, 6, 7, 8, 10, 11, 12, 13 | 14 | 9 | (1) Female sample  (4) Rural area  (9) Small sample of Latinos  (10) Rural area  (12) Female sample  (14) Higher than average poverty rate |
| 3.2. Are measurements appropriate regarding both the outcome and intervention (or exposure)? | 1, 2, 3, 4, 5, 6, 7, 8, 9, 10, 11, 12, 13, 14 |  |  |  |
| 3.3. Are there complete outcome data? | 1, 2, 3, 5, 6, 7, 8, 9, 11, 12, 13, 14 |  | 4 |  |
| 3.4. Are the confounders accounted for in the design and analysis? | 1, 2, 3, 4, 5, 6, 7, 8, 10, 13, 14 | 11, 12 | 9 | (9) Regression considered baseline scores |
| 3.5. During the study period, is the intervention administered (or exposure occurred) as intended? | 1, 2, 3, 4, 5, 6, 7, 8, 9, 10, 11, 12, 13, 14 |  |  |  |

**Note**: (**1**) Choi et al. (2017); (**2**) Copp & Johnson (2015); (**3**) Exner-Cortens et al. (2013); (**4**) Foshee et al. (2013); (**5**) Mulla et al. (2020); (**6**) Mumford et al. (2019b); (**7**) Nahapetyan et al. (2014); (**8**) Pierce (2017); (**9**) Reyes et al. (2017); (**10**) Reyes et al. (2018) ; (**11**) Shorey et al. (2015); (**12**) Smith et al. (2003); (**13**) Taylor et al. (2017); (**14**) Taylor & Sullivan (2017).
