# Supplementary material for: Longitudinal Associations Between Adolescent Dating Violence Victimization and Adverse Outcomes: A Systematic Review
Source: Trauma Violence Abuse. 2023 May 25;25(2):1265–77. doi: 10.1177/15248380231174504 (PMC10913345; doi:10.1177/15248380231174504)
Supplement: sj-docx-2-tva-10.1177_15248380231174504 – Supplemental material for Longitudinal Associations Between Adolescent Dating Violence Victimization and Adverse Outcomes: A Systematic Review [file sj-docx-2-tva-10.1177_15248380231174504.docx]

**Supplementary Material**

## Search Strategy

### Databases.

An electronic search for peer-reviewed and grey literature was conducted between November and December 2020, covering the period between January 2000 and December 2020. Searches were mostly conducted in English, but also in Spanish in relevant databases, as indicated below below.

The databases searched for this review were:

- Pubmed/Medline
- IBSS
- Global Index Medicus WHO
- Proquest One Literature
- JSTOR
- PsycINFO
- LILACS
- SciELO
- Google Scholar

After discussion with the team, the following Spanish journals were also searched:

- PSICOTHEMA
- ANALES DE PSICOLOGÍA
- REVISTA ESPAÑOLA DE INVESTIGACIÓN CRIMINOLÓGICA
- BOLETIN CRIMINOLÓGICO
- THE SPANISH JOURNAL OF PSYCHOLOGY
- REVISTA DE PSICOTERAPIA
- ANUARIO DE PSICOLOGÍA
- ANUARIO DE PSICOLOGÍA JURÍDICA

###

### Search Terms.

The main search terms are listed below. Terms were grouped in five categories (i.e., A, B, C, D, E) and combined under the condition “AND” in the previously specified databases’ search engines, mainly in abstract&title.

Search Option 1: Search Terms.

| A | Adolescen* OR teen* OR youth OR young |
| --- | --- |
| B | partner OR dating OR romantic OR couple |
| C | violen* OR victim* OR survivor OR rape OR abuse OR aggress* |
| D | effects OR consequences OR outcomes OR internalising OR externalising OR symptom* OR mental health OR wellbeing OR depress* OR anxiety OR self-harm OR anger OR suicid* OR attention deficit OR hyperactivity OR conduct disorder OR opposition OR defiance OR aggress OR proactive OR reactive OR post-traumatic stress disorder OR PTSD OR self-esteem OR substance abuse OR eating disorder OR anorexia OR bulimia OR binge-eating OR internalizing OR externalizing OR well-being OR stress OR disorder OR drug OR alcohol OR smoking OR cigarette OR marihuana OR hypersexualization OR hypersexualisation OR prostitution OR psychotic OR compulsive OR intimate partner violence OR resilien* |
| E | Longitudinal OR cohort OR follow-up OR panel |

It was not possible to implement the above combination of terms in all the database interfaces due to language (i.e.., SciELO, Spanish journals), word count (i.e., JSTOR), or search lines (i.e., LILACS) restrictions. For this reason, the main search terms were either translated into Spanish or adapted in number of terms or combinations for certain databases, always taking the terms in Search Option 1 as a reference and without adding any new or different constructs. The four adapted searches can be seen below.

*Search Option 2*: *JSTOR.*

| A | adolescent dating violence OR teen dating violence |
| --- | --- |
| D | consequences OR effect OR outcomes OR internalising OR externalising / consequences OR effect OR outcomes OR internalizing OR externalizing |
| E | Longitudinal OR cohort OR follow-up OR panel |

*Search Option 3*: *LILACS.*

| A | adolescent dating violence OR teen dating violence |
| --- | --- |
| D | effects OR consequences OR outcomes OR internalising OR externalising OR symptom* OR mental health OR wellbeing OR depress* OR anxiety OR self-harm OR anger OR suicid* OR attention deficit OR hyperactivity OR conduct disorder OR opposition OR defiance OR aggress OR proactive OR reactive OR post-traumatic stress disorder OR PTSD OR self-esteem OR substance abuse OR eating disorder OR anorexia OR bulimia OR binge-eating OR internalizing OR externalizing OR well-being OR stress OR disorder OR drug OR alcohol OR smoking OR cigarette OR marihuana OR hypersexualization OR hypersexualisation OR prostitution OR psychotic OR compulsive OR intimate partner violence OR resilien* |
| E | Longitudinal OR cohort OR follow-up OR panel |

*Search Option 4: SCIELO (Spanish).*

| A | Adolescen* O joven O puber* O juventud |
| --- | --- |
| B | pareja O cita O romantic* |
| C | violen* O victim* O superviviente O violación O abuso O agres* |
| D | Efecto* O consecuencia* O resultado* O internaliz* O externaliz* O síntoma* O salud mental O bienestar O depres* O ansiedad O autolesión O ira O suicidio O déficit de atención O hiperactividad O trastorno de conducta O oposición O desafío O agres* O proactivo O reactivo O trastorno de estrés postraumático O TEPT O autoestima O abuso de sustancias O trastorno alimentario O anorexia O bulimia O atracones O estrés O trastorno O drogas O alcohol O tabaquismo O cigarrillo O fuma* O marihuana O hipersexualiza* O prostitución O psicótic* O compuls* O violencia de pareja O violencia de género O violencia machista O resilienc* |
| E | Cohorte O longitudinal O panel O seguimiento |

Despite the challenges associated with systematic scientific literature retrieval in Google Scholar we included this web search engine in the search strategy given that, as a unified interface, it provided potentially relevant results that were not displayed in the other selected databases. After consulting with two researchers with vast experience in using Google Scholar search for systematic reviews, we formulated a systematic search strategy. The strategy consisted of generating several term combinations based on the terms in Search Option 1, both in English and in Spanish, and exporting references for each of the searches until the results listed by page were no longer relevant to the review’s topic. The exported references were systematically screened along with the references obtained from the other databases.

*Search Option 5*: *Google Scholar.*

| Must contain | One of the words | Until page |
| --- | --- | --- |
| adolescent dating violence | longitudinal, OR cohort, OR follow OR up | 35 |
| adolescent dating violence | consequences, OR outcomes, OR effects | 15 |
| teen dating violence | longitudinal, OR cohort, OR follow OR up | 35 |
| teen dating violence | consequences, OR outcomes, OR effects | 30 |
| adolescente, violencia, pareja | longitudinal, OR cohorte, OR consecuencias, OR trastorno | 15 |

All search options used in each database are summarized below.

Summary of search options used in each database.

| Database | Search Option(s) |
| --- | --- |
| Pubmed | 1 |
| IBSS | 1 |
| Global Index Medicus WHO | 1 |
| Proquest One Literature | 1 |
| PsycINFO | 1 |
| JSTOR | 2 |
| LILACS | 3 |
| SCIELO | 1,4 |
| Google Scholar | 5 |
