# Supplementary material for: Longitudinal Associations Between Adolescent Dating Violence Victimization and Adverse Outcomes: A Systematic Review
Source: Trauma Violence Abuse. 2023 May 25;25(2):1265–77. doi: 10.1177/15248380231174504 (PMC10913345; doi:10.1177/15248380231174504)
Supplement: sj-docx-4-tva-10.1177_15248380231174504 – Supplemental material for Longitudinal Associations Between Adolescent Dating Violence Victimization and Adverse Outcomes: A Systematic Review [file sj-docx-4-tva-10.1177_15248380231174504.docx]

**Supplementary Material**

**Sampling Strategies and Modes of Survey Administration Used in the Included Publications**

|  | Sampling strategy | Mode of survey administration |
| --- | --- | --- |
| Choi et al. (2017) | Convenience | Self-administered |
| Copp & Johnson (2015) | Probability | Face-to-face interviews & self-administered |
| Exner-Cortens et al. (2013) | Convenience | Self-administered (A-CASI) |
| Foshee et al. (2013) | Convenience | Self-administered |
| Mulla et al. (2020) | Probability | Self-administered |
| Mumford et al. (2019b) | Probability | Self-administered (CAPI) |
| Nahapetyan et al. (2014) | Probability | Self-administered (CAPI) |
| Pierce (2017) | Convenience | Self-administered (A-CASI) |
| Reyes et al. (2017) | Convenience | Face-to-face interviews |
| Reyes et al. (2018) | Probability | Face-to-face interviews |
| Shorey et al. (2015) | Convenience | Self-administered |
| Smith et al. (2003) | Convenience | Self-administered |
| Taylor et al. (2017) | Probability | Self-administered (CAPI) |
| Taylor & Sullivan (2017) | Probability | Self-administered (A-CASI) |

**Abbreviations**. **A-CASI**: Audio Computer-Assisted Sel-Interviewing; **CAPI**: Computer-Assisted Self-Interviewing.
